# Crystal structure and Hirshfeld surface analysis of ethyl 2-{[4-ethyl-5-(quinolin-8-yloxymeth­yl)-4*H*-1,2,4-triazol-3-yl]sulfan­yl}acetate

**DOI:** 10.1107/S205698901700041X

**Published:** 2017-01-13

**Authors:** Rawia Imane Bahoussi, Ahmed Djafri, Abdelkader Chouaih, Ayada Djafri, Fodil Hamzaoui

**Affiliations:** a Laboratory of Technology and Solid Properties (LTPS), Abdelhamid Ibn Badis University, BP 227 Mostaganem 27000, Algeria; b Centre de Recherche Scientifique et Technique en Analyses, Physico-chimiques (CRAPC), BP 384-Bou-Ismail-RP 42004, Tipaza, Algeria; c Laboratory of Applied Organic Synthesis(LSOA), Department of Chemistry, Faculty of Sciences, University of Oran 1 – Ahmed Ben Bella, 31000 Oran, Algeria

**Keywords:** crystal structure, hydrogen bonding, π–π stacking, 1,2,4-triazole, Hirshfeld surface analysis

## Abstract

In the title compound, C_18_H_20_N_4_O_3_S, the 1,2,4-triazole ring is twisted with respect to the mean plane of quinoline moiety at 65.24 (4)°. In the crystal, mol­ecules are linked by weak C—H⋯O and C—H⋯N hydrogen bonds, forming the three-dimensional supra­molecular packing. π–π stacking between the quinoline ring systems of neighbouring mol­ecules is also observed, the centroid-to-centroid distance being 3.6169 (6) Å. Hirshfeld surface (HS) analyses were performed.

## Abstract 2

In the title compound, the 1,2,4-triazole ring is twisted with respect to the mean plane of quinoline moiety at 65.24 (4)°. In the crystal, mol­ecules are linked by weak C—H⋯O and C—H⋯N hydrogen bonds.

## Chemical context   

Quinoline derivatives are a very important class of nitro­gen-containing heterocycles, which display a broad range of biological activities (Srikanth *et al.*, 2010 ▸). In addition, quinolines have suitable electron mobility and other important properties which are crucial for their use in organic light-emitting diodes (OLEDs) (Chen & Shi, 1998 ▸; Kulkarni *et al.*, 2004 ▸). They are also used in the synthesis of mol­ecules having non-linear optical properties (MacDiarmid *et al.*, 1997 ▸; Epstein, 1997 ▸). The 1,2,4-triazole ring is also a major five-membered heterocyclic ring, which serves as the core component of many substances that display a wide range of biological activities (Mathew *et al.*, 2007 ▸; Pelz *et al.*, 2001 ▸). This heterocycle is an important structural motif in the design of new drugs (Catarzi *et al.*, 2004 ▸). Here we report the mol­ecular and crystal structure of the title 1,2,4-triazole derivative.
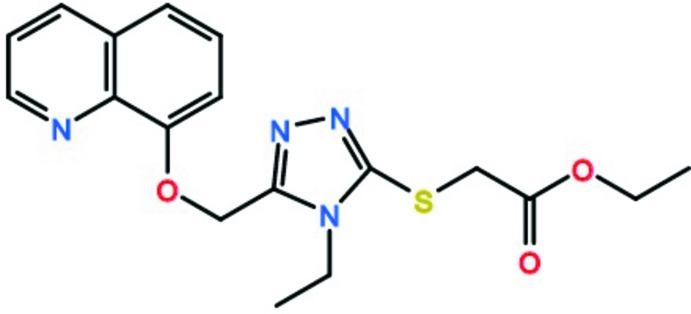


## Structural commentary   

**Figure 1:**
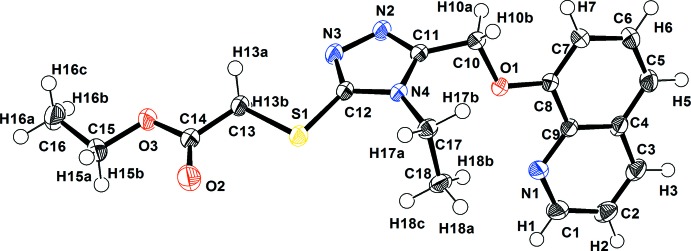
The mol­ecular structure of the title mol­ecule, showing the atomic numbering scheme (displacement ellipsoids are drawn at the 65% probability level). H atoms are shown as small spheres of arbitrary radii.

The mol­ecular structure with atomic numbering scheme for the title compound is given in Fig. 1 ▸. The geometric parameters of the ester group are within normal ranges. Likewise, the S1—C12 and S1—C13 distances, being of 1.7480 (9) and 1.8082 (10) Å, are in agreement with single thio­ether C—S bonds. The C12—S1 bond is shorter than C13—S1 due to the presence of a delocalized π-electronic system throughout the triazole ring. The C—C bond lengths in the quinoline moiety are in the range 1.3691 (16)– 1.4328 (12) Å. The bond lengths are consistent with previous studies (Cabrera *et al.*, 2015 ▸; Sunitha *et al.*, 2015 ▸). The ethyl group C—C bond lengths are in the range 1.5083 (13)–1.5232 (13) Å and are consistent with previously reported values (Alshawi *et al.*, 2015 ▸). The C1—N1, C11—N2 and C12—N3 bonds have double-bond character with bond lengths of 1.3222 (13), 1.3142 (12) and 1.3205 (12) Å, respectively, while the other C—N bonds in the triazole and quinoline rings (C9—N1, C11—N4 and C12—N4) have single-bond character with bond lengths of 1.3662 (12), 1.3699 (11) and 1.3647 (11) Å, respectively. The C14–O3 bond length [1.3326 (12) Å] is notably shorter than the normal C—O single bond (1.427 Å; Wan *et al.*, 2008 ▸) due to conjugation. The C15—O3 bond length [1.4605 (12) Å)] is normal for a C—O single bond. The 1,2,4-triazole ring is almost planar (r.m.s. deviation for the non-H atoms = 0.172 Å) and the ethyl acetate fragment adopts a fully extended conformation. The quinoline ring system and the 1,2,4 triazole ring are not coplanar but inclined to one another by 65.24 (4)°.

## Supra­molecular features   

**Figure 2:**
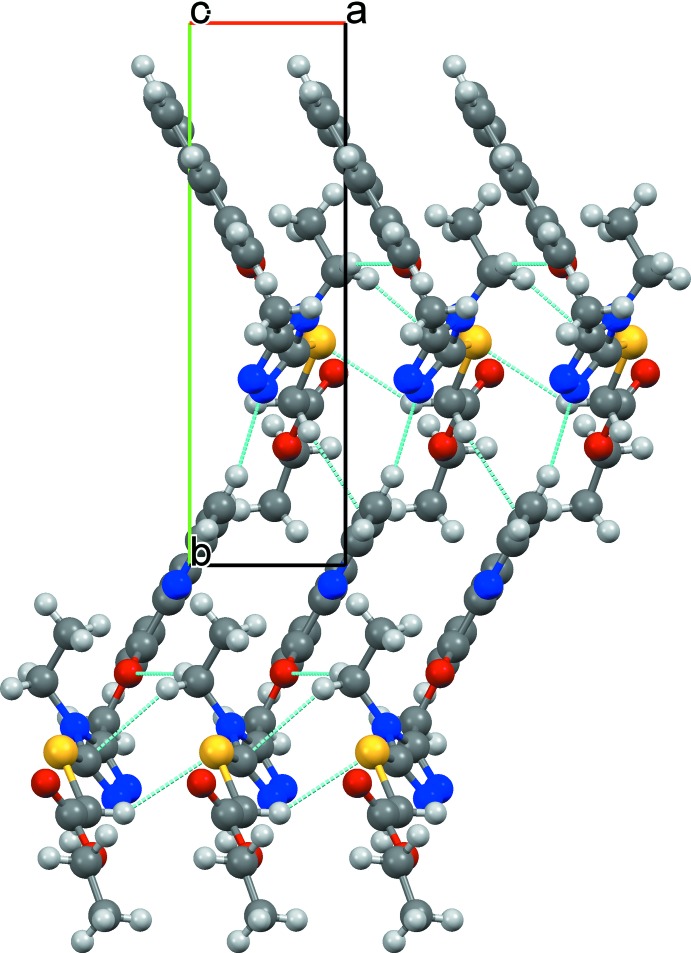
Crystal packing diagram of the title compound viewed along the *c* axis with hydrogen bonds shown as dashed lines.

In the crystal, weak C—H⋯O and C—H⋯N hydrogen bonds (Table 1 ▸, Fig. 2 ▸) link the mol­ecules into a three dimensional supra­molecular architecture. π–π stacking involving the quinoline rings is also observed, with the intercentroid distance being 3.6169 (6) Å.

## Hirshfeld surface analysis   

**Figure 3:**
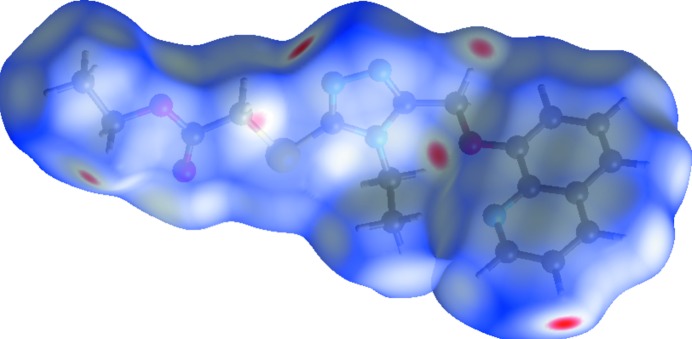
HS mapped over *d*_norm_.

**Figure 4:**
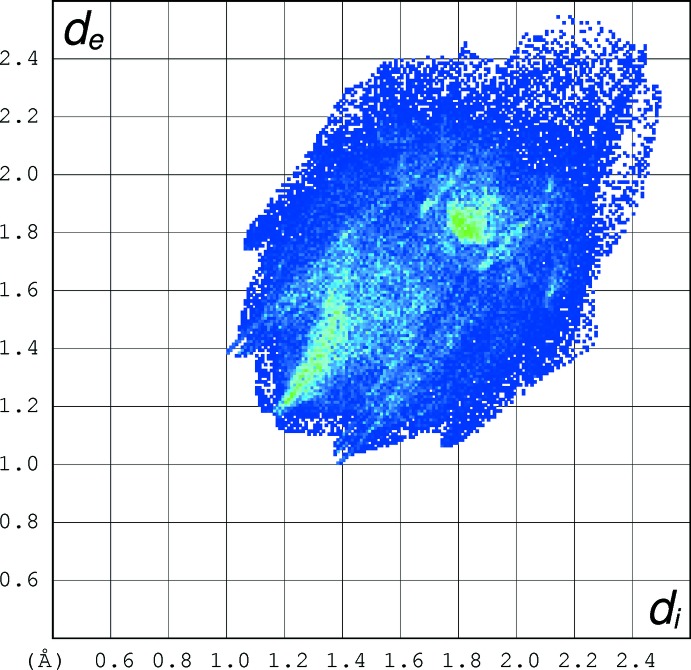
The two-dimensional fingerprint plot of the title mol­ecule.

To understand the different inter­actions and contacts in the crystal structure, it is necessary to represent Hirshfeld surface (HS) and generate fingerprint plots which provide qu­anti­tative information for each inter­molecular inter­action. In order to highlight all intra- and inter­molecular inter­actions, HS analyses were performed and fingerprint plots were drawn using *Crystal Explorer* (Wolff *et al.*, 2007 ▸). The three-dimensional Hirshfeld surface generated for the structure of the title crystal is presented in Fig. 3 ▸, which shows surfaces that have been mapped over a *d*_norm_ range of −0.191 to 1.247 Å. The large deep-red spots on the *d*_norm_ HS indicate the close-contact inter­actions, which are mainly responsible for significant hydrogen-bonding contacts. The 2D fingerprint plot is depicted in Fig. 4 ▸. This indicates that the most important contacts on the surface, which are necessary for organic mol­ecules, are the H⋯H contacts with a percent contribution of 47.7% to the HS area of the title mol­ecule.

## Synthesis and crystallization   

**Figure 5:**
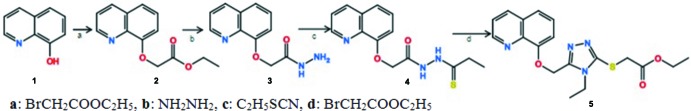
Chemical pathway showing the formation of the title compound.

The synthesis of the title compound was performed according to the scheme in Fig. 5 ▸. Eth­yl(quinoline-8-yl­oxy)acetate (2) was synthesized by condensation of 8-hy­droxy­quinoline (0.01 mol) (1) with ethyl bromo­acetate (0.01 mol) in dry acetone for 12 h in the presence of anhydrous K_2_CO_3_. A mixture of compound (2) (0.01 mol) and hydrazine hydrate (0.02 mol) in ethanol was refluxed for 1 h. After cooling, the resulting solid was washed, dried and recrystallized from ethanol to afford 2-(quinolin-8-yl­oxy)acetohydrazide (3). Compound (3), on reaction with ethyl thio­cyanate gave (quinolin-8-yl­oxy)-acetic acid *N*′-thio­propionyl-hydrazide (4). To a solution of compound (4) (0.01 mol) in absolute ethanol and (2 eq) of anhydrous CH_3_COONa, ethyl bromo­acetate (0.01 mol) was added. After refluxing for 12 h, the formed precipitate was filtered off and recrystallized from ethanol to give the title compound (5) with moderate yield (75%, m.p. 284 K). Single crystals of the title compound suitable for X-ray diffraction were obtained from ethanol solution.

IR (KBr, cm^−1^): 2967(CH3), 1730 (C=O), 1618–1486 (C=C), 1429 (C=N), 1174 (N—N), 819 (C—S). ^1^H NMR, (CDCl_3_, 300 MHz) δ (p.p.m.) *J* (Hz): 1.12 (*t*, 3H, *J* = 7.20 Hz, OCH_2_CH_3_), 1.27 (*t*, 3H, *J* = 7.21 Hz, NCH_2_CH_3_), 4.00 (*s*, 2H, S—CH_2_), 4.07 (*q*, 2H, *J* = 7.17 Hz, N—CH_2_), 4.16 (*q*, 2H, *J* = 7.25 Hz, O—CH_2_CH_3_), 5.50 (*s*, 2H, O—CH_2_), 7.28–7.34 (*m*, 4H, Ar—H), 7.03 (*dd*, 1H, *J* = 1.56 Hz, *J* = 8.26 Hz, Ar—H), 8.81 (*dd*, 1H, *J* = 1.56 Hz, *J* = 4.13 Hz, Ar—H). ^13^C NMR, (CDCl_3_, 300 MHz) δ (p.p.m.): 14.03 (CH_3_), 15.35(CH_3_), 35.02(N—CH_2_), 39.89 (S—CH_2_), 61.41(O—CH_2_), 62.09 (O—CH_2_CH_3_), 110.80, 121.08, 121.75, 126.72, 129.49, 136.02, 1140.15, 149.41, 150.75, 151.45, 153.04, 168.26 (C=O).

## Refinement   

Crystal data, data collection and structure refinement details are summarized in Table 2 ▸. H atoms in the title compound were placed in calculated positions (C—H = 0.96–1.08 Å) and allowed to ride on their parent atoms with *U*_iso_(H) = 1.5*U*_eq_(C) for methyl H atoms and 1.2*U*_eq_(C) for other H atoms.

## Supplementary Material

Crystal structure: contains datablock(s) I, global. DOI: 10.1107/S205698901700041X/xu5898sup1.cif

Structure factors: contains datablock(s) I. DOI: 10.1107/S205698901700041X/xu5898Isup2.hkl

Supporting information file. DOI: 10.1107/S205698901700041X/xu5898Isup3.cml

CCDC reference: 1477670

Additional supporting information:  crystallographic information; 3D view; checkCIF report

## Figures and Tables

**Table 1:** Hydrogen-bond geometry (Å, °)

*D*—H⋯*A*	*D*—H	H⋯*A*	*D*⋯*A*	*D*—H⋯*A*
C2—H2⋯N3^i^	0.95	2.51	3.4444 (14)	167
C10—H10*A*⋯O2^ii^	0.99	2.52	3.4637 (13)	159
C15—H15*B*⋯N2^iii^	0.99	2.58	3.4636 (14)	148
C17—H17*B*⋯O1^iv^	0.99	2.50	3.4481 (12)	161

Symmetry codes: (i) 
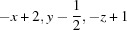
; (ii) 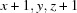; (iii) 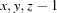; (iv) 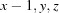.

**Table 2:** Experimental details

Crystal data	
Chemical formula	C_18_H_20_N_4_O_3_S
*M*_r_	372.44
Crystal system, space group	Monoclinic, *P*2_1_
Temperature (K)	100
*a*, *b*, *c* (Å)	4.0880 (3), 21.2246 (15), 10.2037 (7)
β (°)	99.407 (3)
*V* (Å^3^)	873.43 (11)
*Z*	2
Radiation type	Mo *K*α
μ (mm^−1^)	0.21
Crystal size (mm)	0.55 × 0.10 × 0.09

Data collection	
Diffractometer	Nonius KappaCCD
Absorption correction	ψ scan (North *et al.*, 1968 ▸)
*T*_min_, *T*_max_	0.973, 0.981
No. of measured, independent and observed [*I* > 2σ(*I*)] reflections	75160, 14165, 12491
*R*_int_	0.045
(sin θ/λ)_max_ (Å^−1^)	1.002

Refinement	
*R*[*F* ^2^ > 2σ(*F* ^2^)], *wR*(*F* ^2^), *S*	0.036, 0.094, 1.06
No. of reflections	14165
No. of parameters	237
No. of restraints	1
H-atom treatment	H-atom parameters constrained
Δρ_max_, Δρ_min_ (e Å^−3^)	0.41, −0.25
Absolute structure	Flack *x* determined using 5451 quotients [(*I* ^+^)−(*I* ^−^)]/[(*I* ^+^)+(*I* ^−^)] (Parsons *et al.*, 2013 ▸)
Absolute structure parameter	0.253 (3)

Computer programs: *KappaCCD* Nonius (Nonius, 1998 ▸), *DENZO* and *SCALEPACK* (Otwinowski & Minor, 1997 ▸), *SHELXS97* (Sheldrick, 2008 ▸), *SHELXL2014* (Sheldrick, 2015 ▸), *ORTEP-3 for Windows* (Farrugia, 2012 ▸) and *Mercury* (Macrae *et al.*, 2006 ▸).

## supplementary crystallographic information

### Crystal data

**Table:**

C_18_H_20_N_4_O_3_S	*F*(000) = 392
*M**_r_* = 372.44	*D*_x_ = 1.416 Mg m^−^^3^
Monoclinic, *P*2_1_	Mo *K*α radiation, λ = 0.71073 Å
*a* = 4.0880 (3) Å	Cell parameters from 100 reflections
*b* = 21.2246 (15) Å	θ = 2–29°
*c* = 10.2037 (7) Å	µ = 0.21 mm^−^^1^
β = 99.407 (3)°	*T* = 100 K
*V* = 873.43 (11) Å^3^	Prism, yellow
*Z* = 2	0.55 × 0.10 × 0.09 mm

### Data collection

**Table:**

Nonius KappaCCD diffractometer	12491 reflections with *I* > 2σ(*I*)
θ/2θ scans	*R*_int_ = 0.045
Absorption correction: ψ scan (North *et al.*, 1968)	θ_max_ = 45.4°, θ_min_ = 1.9°
*T*_min_ = 0.973, *T*_max_ = 0.981	*h* = −7→7
75160 measured reflections	*k* = −42→42
14165 independent reflections	*l* = −20→20

### Refinement

**Table:**

Refinement on *F*^2^	Hydrogen site location: inferred from neighbouring sites
Least-squares matrix: full	H-atom parameters constrained
*R*[*F*^2^ > 2σ(*F*^2^)] = 0.036	*w* = 1/[σ^2^(*F*_o_^2^) + (0.0538*P*)^2^ + 0.0281*P*] where *P* = (*F*_o_^2^ + 2*F*_c_^2^)/3
*wR*(*F*^2^) = 0.094	(Δ/σ)_max_ < 0.001
*S* = 1.06	Δρ_max_ = 0.41 e Å^−^^3^
14165 reflections	Δρ_min_ = −0.25 e Å^−^^3^
237 parameters	Absolute structure: Flack *x* determined using 5451 quotients [(*I*^+^)-(*I*^-^)]/[(*I*^+^)+(*I*^-^)] (Parsons *et al.*, 2013)
1 restraint	Absolute structure parameter: 0.253 (3)

### Special details

**Table:**

Geometry. All esds (except the esd in the dihedral angle between two l.s. planes) are estimated using the full covariance matrix. The cell esds are taken into account individually in the estimation of esds in distances, angles and torsion angles; correlations between esds in cell parameters are only used when they are defined by crystal symmetry. An approximate (isotropic) treatment of cell esds is used for estimating esds involving l.s. planes.

### Fractional atomic coordinates and isotropic or equivalent isotropic displacement parameters (Å^2^)

**Table:**

	*x*	*y*	*z*	*U*_iso_*/*U*_eq_	
N1	0.9172 (2)	0.02368 (4)	0.62203 (8)	0.01693 (13)	
C1	1.0744 (3)	−0.03017 (5)	0.61284 (11)	0.02013 (16)	
H1	1.1114	−0.0430	0.5272	0.024*	
C2	1.1915 (3)	−0.07009 (5)	0.72130 (12)	0.02139 (17)	
H2	1.3007	−0.1086	0.7084	0.026*	
C3	1.1431 (3)	−0.05183 (5)	0.84577 (11)	0.01943 (16)	
H3	1.2234	−0.0772	0.9209	0.023*	
C4	0.9726 (2)	0.00508 (4)	0.86158 (9)	0.01551 (13)	
C5	0.9089 (3)	0.02636 (5)	0.98684 (10)	0.01889 (15)	
H5	0.9840	0.0024	1.0646	0.023*	
C6	0.7390 (3)	0.08139 (5)	0.99544 (9)	0.01881 (15)	
H6	0.6948	0.0950	1.0795	0.023*	
C7	0.6280 (3)	0.11840 (5)	0.88129 (9)	0.01619 (13)	
H7	0.5097	0.1564	0.8890	0.019*	
C8	0.6921 (2)	0.09907 (4)	0.75895 (9)	0.01349 (12)	
C9	0.8639 (2)	0.04117 (4)	0.74566 (9)	0.01360 (12)	
O1	0.6071 (2)	0.13178 (3)	0.64335 (7)	0.01586 (11)	
C10	0.4482 (2)	0.19139 (4)	0.65147 (9)	0.01451 (12)	
H10A	0.5765	0.2178	0.7217	0.017*	
H10B	0.2217	0.1856	0.6720	0.017*	
C11	0.4356 (2)	0.22133 (4)	0.51895 (8)	0.01328 (12)	
N4	0.26324 (19)	0.19754 (3)	0.40309 (7)	0.01236 (10)	
C12	0.3301 (2)	0.23854 (4)	0.30789 (8)	0.01341 (12)	
N3	0.5287 (2)	0.28427 (4)	0.36011 (8)	0.01678 (13)	
N2	0.5964 (2)	0.27277 (4)	0.49637 (8)	0.01664 (12)	
S1	0.17236 (6)	0.22776 (2)	0.13959 (2)	0.01548 (4)	
C13	0.3319 (2)	0.30058 (4)	0.08308 (9)	0.01582 (13)	
H13A	0.2351	0.3369	0.1241	0.019*	
H13B	0.5758	0.3021	0.1094	0.019*	
C14	0.2422 (2)	0.30408 (4)	−0.06611 (9)	0.01528 (13)	
O2	0.0756 (3)	0.26564 (5)	−0.13459 (9)	0.02499 (16)	
O3	0.3753 (2)	0.35520 (4)	−0.11198 (8)	0.02008 (13)	
C15	0.3339 (3)	0.36198 (5)	−0.25619 (10)	0.01947 (16)	
H15A	0.0978	0.3570	−0.2961	0.023*	
H15B	0.4652	0.3296	−0.2945	0.023*	
C16	0.4544 (3)	0.42708 (6)	−0.28335 (12)	0.02388 (19)	
H16A	0.4462	0.4326	−0.3792	0.036*	
H16B	0.6831	0.4324	−0.2379	0.036*	
H16C	0.3122	0.4586	−0.2506	0.036*	
C17	0.0521 (2)	0.14137 (4)	0.38461 (9)	0.01435 (12)	
H17A	−0.1426	0.1503	0.3158	0.017*	
H17B	−0.0302	0.1322	0.4687	0.017*	
C18	0.2296 (3)	0.08330 (4)	0.34364 (10)	0.01737 (14)	
H18A	0.0760	0.0475	0.3327	0.026*	
H18B	0.4194	0.0734	0.4124	0.026*	
H18C	0.3076	0.0916	0.2594	0.026*	

### Atomic displacement parameters (Å^2^)

**Table:**

	*U*^11^	*U*^22^	*U*^33^	*U*^12^	*U*^13^	*U*^23^
N1	0.0207 (3)	0.0149 (3)	0.0152 (3)	0.0034 (2)	0.0031 (2)	0.0000 (2)
C1	0.0242 (4)	0.0164 (4)	0.0196 (4)	0.0053 (3)	0.0030 (3)	−0.0012 (3)
C2	0.0238 (4)	0.0144 (4)	0.0248 (4)	0.0045 (3)	0.0007 (3)	0.0004 (3)
C3	0.0217 (4)	0.0139 (3)	0.0215 (4)	0.0020 (3)	0.0000 (3)	0.0033 (3)
C4	0.0176 (3)	0.0123 (3)	0.0157 (3)	−0.0008 (2)	0.0000 (2)	0.0022 (2)
C5	0.0236 (4)	0.0184 (4)	0.0140 (3)	−0.0005 (3)	0.0011 (3)	0.0033 (3)
C6	0.0238 (4)	0.0196 (4)	0.0131 (3)	−0.0006 (3)	0.0032 (3)	0.0011 (3)
C7	0.0203 (4)	0.0156 (3)	0.0129 (3)	0.0003 (3)	0.0035 (2)	−0.0001 (2)
C8	0.0161 (3)	0.0118 (3)	0.0125 (3)	0.0002 (2)	0.0022 (2)	0.0009 (2)
C9	0.0157 (3)	0.0113 (3)	0.0136 (3)	−0.0002 (2)	0.0015 (2)	0.0008 (2)
O1	0.0231 (3)	0.0119 (2)	0.0129 (2)	0.0043 (2)	0.0036 (2)	0.00195 (19)
C10	0.0182 (3)	0.0113 (3)	0.0142 (3)	0.0022 (2)	0.0029 (2)	0.0004 (2)
C11	0.0160 (3)	0.0102 (3)	0.0134 (3)	0.0002 (2)	0.0018 (2)	0.0003 (2)
N4	0.0143 (3)	0.0096 (2)	0.0132 (2)	−0.00051 (18)	0.00228 (19)	0.00067 (19)
C12	0.0161 (3)	0.0104 (3)	0.0136 (3)	−0.0003 (2)	0.0020 (2)	0.0013 (2)
N3	0.0229 (4)	0.0119 (3)	0.0148 (3)	−0.0035 (2)	0.0009 (2)	0.0014 (2)
N2	0.0221 (3)	0.0121 (3)	0.0150 (3)	−0.0029 (2)	0.0010 (2)	0.0005 (2)
S1	0.01887 (9)	0.01298 (8)	0.01402 (8)	−0.00283 (6)	0.00099 (6)	0.00105 (7)
C13	0.0207 (4)	0.0117 (3)	0.0144 (3)	−0.0014 (2)	0.0011 (2)	0.0015 (2)
C14	0.0183 (3)	0.0127 (3)	0.0147 (3)	−0.0004 (2)	0.0022 (2)	0.0008 (2)
O2	0.0335 (4)	0.0221 (4)	0.0177 (3)	−0.0115 (3)	−0.0007 (3)	−0.0001 (3)
O3	0.0313 (4)	0.0147 (3)	0.0141 (2)	−0.0057 (2)	0.0032 (2)	0.0012 (2)
C15	0.0256 (4)	0.0186 (4)	0.0142 (3)	−0.0015 (3)	0.0034 (3)	0.0018 (3)
C16	0.0293 (5)	0.0200 (4)	0.0235 (4)	0.0006 (3)	0.0078 (4)	0.0068 (4)
C17	0.0139 (3)	0.0121 (3)	0.0173 (3)	−0.0022 (2)	0.0032 (2)	−0.0002 (2)
C18	0.0204 (4)	0.0112 (3)	0.0206 (4)	−0.0017 (2)	0.0038 (3)	−0.0023 (3)

### Geometric parameters (Å, º)

**Table:**

N1—C1	1.3222 (13)	N4—C12	1.3647 (11)
N1—C9	1.3662 (12)	N4—C17	1.4660 (11)
C1—C2	1.4137 (15)	C12—N3	1.3205 (12)
C1—H1	0.9500	C12—S1	1.7480 (9)
C2—C3	1.3728 (17)	N3—N2	1.3941 (12)
C2—H2	0.9500	S1—C13	1.8082 (10)
C3—C4	1.4168 (14)	C13—C14	1.5083 (13)
C3—H3	0.9500	C13—H13A	0.9900
C4—C9	1.4183 (12)	C13—H13B	0.9900
C4—C5	1.4192 (14)	C14—O2	1.2092 (13)
C5—C6	1.3691 (16)	C14—O3	1.3326 (12)
C5—H5	0.9500	O3—C15	1.4605 (12)
C6—C7	1.4166 (14)	C15—C16	1.5078 (16)
C6—H6	0.9500	C15—H15A	0.9900
C7—C8	1.3792 (12)	C15—H15B	0.9900
C7—H7	0.9500	C16—H16A	0.9800
C8—O1	1.3638 (11)	C16—H16B	0.9800
C8—C9	1.4328 (12)	C16—H16C	0.9800
O1—C10	1.4309 (11)	C17—C18	1.5232 (13)
C10—C11	1.4872 (12)	C17—H17A	0.9900
C10—H10A	0.9900	C17—H17B	0.9900
C10—H10B	0.9900	C18—H18A	0.9800
C11—N2	1.3142 (12)	C18—H18B	0.9800
C11—N4	1.3699 (11)	C18—H18C	0.9800
			
C1—N1—C9	116.95 (9)	N3—C12—N4	111.28 (8)
N1—C1—C2	124.67 (10)	N3—C12—S1	126.62 (7)
N1—C1—H1	117.7	N4—C12—S1	122.08 (7)
C2—C1—H1	117.7	C12—N3—N2	106.41 (7)
C3—C2—C1	118.26 (9)	C11—N2—N3	107.27 (8)
C3—C2—H2	120.9	C12—S1—C13	96.14 (4)
C1—C2—H2	120.9	C14—C13—S1	108.85 (6)
C2—C3—C4	119.60 (9)	C14—C13—H13A	109.9
C2—C3—H3	120.2	S1—C13—H13A	109.9
C4—C3—H3	120.2	C14—C13—H13B	109.9
C3—C4—C9	117.29 (9)	S1—C13—H13B	109.9
C3—C4—C5	122.68 (9)	H13A—C13—H13B	108.3
C9—C4—C5	120.03 (9)	O2—C14—O3	124.76 (9)
C6—C5—C4	119.93 (9)	O2—C14—C13	124.82 (9)
C6—C5—H5	120.0	O3—C14—C13	110.41 (8)
C4—C5—H5	120.0	C14—O3—C15	116.57 (8)
C5—C6—C7	121.20 (9)	O3—C15—C16	106.73 (9)
C5—C6—H6	119.4	O3—C15—H15A	110.4
C7—C6—H6	119.4	C16—C15—H15A	110.4
C8—C7—C6	119.76 (9)	O3—C15—H15B	110.4
C8—C7—H7	120.1	C16—C15—H15B	110.4
C6—C7—H7	120.1	H15A—C15—H15B	108.6
O1—C8—C7	124.90 (8)	C15—C16—H16A	109.5
O1—C8—C9	114.47 (7)	C15—C16—H16B	109.5
C7—C8—C9	120.63 (8)	H16A—C16—H16B	109.5
N1—C9—C4	123.21 (8)	C15—C16—H16C	109.5
N1—C9—C8	118.36 (8)	H16A—C16—H16C	109.5
C4—C9—C8	118.42 (8)	H16B—C16—H16C	109.5
C8—O1—C10	117.00 (7)	N4—C17—C18	113.36 (7)
O1—C10—C11	105.89 (7)	N4—C17—H17A	108.9
O1—C10—H10A	110.6	C18—C17—H17A	108.9
C11—C10—H10A	110.6	N4—C17—H17B	108.9
O1—C10—H10B	110.6	C18—C17—H17B	108.9
C11—C10—H10B	110.6	H17A—C17—H17B	107.7
H10A—C10—H10B	108.7	C17—C18—H18A	109.5
N2—C11—N4	110.87 (8)	C17—C18—H18B	109.5
N2—C11—C10	124.77 (8)	H18A—C18—H18B	109.5
N4—C11—C10	124.31 (8)	C17—C18—H18C	109.5
C12—N4—C11	104.16 (7)	H18A—C18—H18C	109.5
C12—N4—C17	127.54 (8)	H18B—C18—H18C	109.5
C11—N4—C17	128.29 (7)		
			
C9—N1—C1—C2	−0.45 (17)	O1—C10—C11—N4	63.40 (11)
N1—C1—C2—C3	−0.74 (19)	N2—C11—N4—C12	−0.21 (10)
C1—C2—C3—C4	1.42 (17)	C10—C11—N4—C12	−178.06 (8)
C2—C3—C4—C9	−0.95 (15)	N2—C11—N4—C17	−179.65 (8)
C2—C3—C4—C5	178.93 (11)	C10—C11—N4—C17	2.50 (14)
C3—C4—C5—C6	−179.14 (10)	C11—N4—C12—N3	0.06 (10)
C9—C4—C5—C6	0.74 (15)	C17—N4—C12—N3	179.50 (9)
C4—C5—C6—C7	−0.84 (16)	C11—N4—C12—S1	178.60 (7)
C5—C6—C7—C8	−0.22 (16)	C17—N4—C12—S1	−1.95 (13)
C6—C7—C8—O1	−177.76 (9)	N4—C12—N3—N2	0.10 (11)
C6—C7—C8—C9	1.37 (14)	S1—C12—N3—N2	−178.36 (7)
C1—N1—C9—C4	0.96 (15)	N4—C11—N2—N3	0.27 (11)
C1—N1—C9—C8	−179.58 (9)	C10—C11—N2—N3	178.12 (8)
C3—C4—C9—N1	−0.28 (14)	C12—N3—N2—C11	−0.22 (11)
C5—C4—C9—N1	179.84 (9)	N3—C12—S1—C13	−6.16 (10)
C3—C4—C9—C8	−179.73 (9)	N4—C12—S1—C13	175.53 (8)
C5—C4—C9—C8	0.38 (14)	C12—S1—C13—C14	178.01 (7)
O1—C8—C9—N1	−1.71 (12)	S1—C13—C14—O2	3.55 (14)
C7—C8—C9—N1	179.08 (9)	S1—C13—C14—O3	−175.82 (7)
O1—C8—C9—C4	177.77 (8)	O2—C14—O3—C15	−4.60 (16)
C7—C8—C9—C4	−1.44 (13)	C13—C14—O3—C15	174.77 (9)
C7—C8—O1—C10	1.90 (14)	C14—O3—C15—C16	171.18 (10)
C9—C8—O1—C10	−177.28 (8)	C12—N4—C17—C18	83.62 (11)
C8—O1—C10—C11	170.64 (8)	C11—N4—C17—C18	−97.06 (11)
O1—C10—C11—N2	−114.16 (10)		

### Hydrogen-bond geometry (Å, º)

**Table:**

*D*—H···*A*	*D*—H	H···*A*	*D*···*A*	*D*—H···*A*
C2—H2···N3^i^	0.95	2.51	3.4444 (14)	167
C10—H10*A*···O2^ii^	0.99	2.52	3.4637 (13)	159
C15—H15*B*···N2^iii^	0.99	2.58	3.4636 (14)	148
C17—H17*B*···O1^iv^	0.99	2.50	3.4481 (12)	161

Symmetry codes: (i) −*x*+2, *y*−1/2, −*z*+1; (ii) *x*+1, *y*, *z*+1; (iii) *x*, *y*, *z*−1; (iv) *x*−1, *y*, *z*.
